# Comparison of routine health-facility (KHIS) versus house-survey (KDHS) estimates of childhood vaccination coverage in Kenya: a cross-sectional study

**DOI:** 10.1136/bmjph-2026-005621

**Published:** 2026-11-11

**Authors:** Cosmas Biwot, Jeanette Dawa, Muema J Mulei, George P Omondi, Damaris Kimonge, Vicky Maiyo, Namu Kimunguyi, Rose Jelang’o, Helen Kiarie, Nyamai Mutono, Loice Achieng Ombajo, S M Thumbi

**Affiliations:** 1 Center for Epidemiological Modelling and Analysis, University of Nairobi, Nairobi, Kenya; 2 Washington State University Paul G Allen School for Global Health, Pullman, Washington, USA; 3 KEMRI-Wellcome Trust Research Programme, Kilifi, Kenya; 4 Kenya Ministry of Health, Nairobi, Kenya; 5 National Vaccines and Immunization Program, Ministry of Health, Nairobi, Kenya; 6 Clinical Medicine and Therapeutics, University of Nairobi, Nairobi, Kenya

**Keywords:** Epidemiologic Methods, methods, Public Health, Vaccination

## Abstract

**Introduction:**

Reliable estimates of effective coverage of health interventions are essential for tracking progress towards health goals and informing policy decisions. In settings where routine health information systems are limited, population-based household surveys are frequently used as reference standards.

**Objective:**

This study compared national and sub-national childhood vaccination coverage estimates from Kenya’s facility-based Health Information System (KHIS) and the 2022 Kenya Demographic Health Survey (KDHS) to assess concordance between the two data sources and to evaluate the suitability of routine data for programmatic decision-making.

**Methods:**

We analysed BCG, pentavalent 1, pentavalent 3 and measles-rubella 1 (MR 1) vaccination data reported by 8178 health facilities to KHIS between March 2020 and March 2022 alongside KDHS 2022 data from 3679 children aged 12–23 months. Coverage estimates were compared at national and county levels using a two-proportion z-test. Absolute differences in coverage were categorised as minimal (<5%), moderate (5–10%) and high (10+%), thresholds previously used in immunisation monitoring as reported by WHO and United Nations International Children’s Emergency Fund coverage comparison.

**Results:**

Nationally, KDHS coverage estimates were higher than KHIS estimates for BCG, pentavalent 1 and MR 1; the absolute differences were all <5% and statistically significant. For pentavalent 3, no significant difference was detected (0.38 percentage points; 95% CI −0.54 to 1.42). Sub-national discrepancies were substantially larger: minimal differences (<5%) were observed in only 15–21 of 47 counties depending on antigen, while high discrepancies (>10%) occurred in 9–14 counties. Only three counties showed absolute differences <5% across all four antigens; several counties showed persistent divergence across antigens.

**Conclusion:**

KHIS yields reasonably concordant national-level estimates of childhood vaccination coverage, but substantial and uneven county-level discrepancies limit its reliability for sub-national decision-making. A differentiated approach is warranted: KHIS can support national trend monitoring and may be usable in counties with demonstrated concordance, whereas counties with persistent divergence require continued survey-based validation and targeted strengthening of routine data systems. The two data sources should not be treated as interchangeable at county level.

**WHAT IS ALREADY KNOWN ON THIS TOPIC**

- Two sources of data used by the ministry of health in Kenya for estimating childhood immunisation coverage are the routine, facility-based Kenya Health Information System (KHIS) and the periodic, population-based Kenya Demographic Health Survey (KDHS). While KHIS data is timely and inexpensive, its accuracy is questionable because of the missing data on children who did not access health facilities, denominator uncertainty and reporting gaps. KDHS is widely considered an accurate population-based benchmark but it is infrequent, arrives late for real-time decisions and faces uncertainty about long-term sustainability amid changes in external donor funding.

**WHAT THIS STUDY ADDS**

- This study has offered a replicable procedure for verifying routine health information against household surveys. It quantifies concordance of childhood immunisation coverage estimates at national and county levels in Kenya, showing that national estimates align within programmatic thresholds while the sub-national estimates were large, antigen- and geographic-specific discrepancies. The method can be applied to other health indicators and offers low- and middle-income countries a country-led approach for determining when routine data can be trusted for programme decisions and where additional validation or system strengthening is needed.

**HOW THIS STUDY MIGHT AFFECT RESEARCH, PRACTICE OR POLICY**

- Policymakers are offered a differentiated approach on reliably using routine data for national trends monitoring, while targeting investments for data quality and improvements in counties with high discrepancies and continuing periodic surveys for validation. Researchers are provided with a replicable framework that can be extended to other routine health indicators. The findings also highlight the need for future research on practical, affordable mechanisms for continuous data-quality assurance within the routine systems.

## Introduction

 Accurate and reliable health indicators are essential for effective public health planning, policy formulation and resource allocation. They provide important information about the health status of populations, aiding the design and execution of interventions, and enable the monitoring of progress towards national and global health targets.^1^ In Kenya, policymakers depend primarily on two major sources of health data: routine health information systems and population-based surveys.^2^ Although both systems aim to generate actionable health intelligence, they differ substantially in their methodologies, scope and frequency.^3^ This raises an important question: to what extent do these two systems produce comparable estimates and how suitable is each for evidence-based decision-making? Understanding their concordance is vital for assessing the validity and utility of different sources of health data for evidence-based decision making.^4,5^

Facility-based routine data collection systems are designed to generate continuous, real-time or near real-time data on key health indicators including disease incidence, service utilisation and service delivery metrics.^6,7^ These systems are essential for the effective functioning of health programmes, as they support timely detection of outbreaks, routine performance monitoring and evaluation of programme impact. However, they often suffer from incomplete reporting, variable data quality and systematic biases related to health-seeking behaviours, geographical access to health facilities and patterns of service utilisation.^8^ Such limitations raise concerns about the extent to which routine health facility data accurately reflect the health status of the entire population, particularly individuals who do not frequently access formal healthcare services.^9^

Population-based household surveys are widely used to complement routine health information systems, particularly in settings where routine reporting may be incomplete or unreliable.^10^ Conducted approximately every 5 years, the Kenya Demographic Health Survey (KDHS) provides nationally representative data across a wide range of health indicators.^2^ The most recent KDHS, released in 2022, was the second survey undertaken since the devolution of health functions to county governments and the seventh overall in the country’s history.^11^ The survey employs a rigorous methodological approach, using the latest national population census data and a two-stage stratified sampling design to select households and individuals.^12^ For this reason, KDHS findings are frequently used to validate or supplement routine health data, providing a more comprehensive picture of population health that includes individuals who may not access formal healthcare services or whose information is not recorded.^13^ Demographic and Health Surveys are widely regarded as the standard for generating population-level health estimates in low- and middle-income countries (LMICs).

Despite their strengths, DHS surveys have notable limitations. Their infrequent implementation prevents real-time monitoring of rapid epidemiological changes or informing timely public health responses.^7,14^ Additionally, their retrospective nature may miss recent programmatic developments or shifts in service delivery, affecting relevance to immediate decision making.^15^ Changes in international aid priorities, particularly reductions in US government funding, have introduced uncertainty around DHS sustainability and frequency.^13,16^

Discrepancies between routine and survey data persist because of differences in population captured, timing of data collection (continuous vs periodic), and measurement practices including indicator definitions, reporting standards and data collection tools can lead to divergent estimates.^17,18^ These inconsistencies pose challenges for health decision-making. Assessing concordance is therefore essential for determining when routine data can be reliably used and when further verification or system strengthening is required. Immunisation coverage offers an ideal context for such assessment: Vaccines are widely delivered, follow standardised schedules and are consistently reported across both systems.

The objective of this study was to compare childhood vaccination coverage estimates from Kenya’s Health Information Systems (KHIS) and the 2022 KDHS at national and sub-national (county) levels and thereby inform more effective use of available health data.

## Methods

### Study design

We conducted a retrospective cross-sectional comparison of childhood vaccination coverage estimates from two data systems in Kenya: facility-based (KHIS) and population-based (KDHS) systems. The design was selected because both KHIS and KDHS report vaccination events over defined periods, potentially allowing estimation of coverage within comparable population cohorts.

### Data sources

We used the publicly available children’s dataset from KDHS 2022, a nationally representative household survey employing a two-stage stratified cluster sampling approach based on the 2019 Kenya Population and Housing Census. The analytical sample included 3679 surviving children aged 12–23 months at the time of the survey.^19^ For children with vaccination cards available at home (75.6%), vaccination status was based on documented records; for the remainder (24.4%), status was determined by caregiver recall.

Data were extracted from KHIS where 8178 health facilities reported immunisation services to KHIS between March 2020 and March 2022.^20–22^ Of these, 6030 were rural, 2136 were urban and 12 were unclassified. Facility ownership included public (69.81%), private (20.46%), faith-based (8.73%) and Non-Governmental Organizations (NGOs) (1.00%).^22^ We aggregated monthly doses administered for BCG, pentavalent 1, pentavalent 3 and measles rubella 1 (MR-1).^23^ Data extraction windows were aligned with the birth months of the youngest and oldest children included in the KDHS 2022 to ensure temporal comparability. These antigens represent early, middle and late schedule doses and are standard benchmarks for assessing the accessibility and utilisation of childhood vaccination services.^24–26^

### Data analysis

Vaccine coverage from KDHS 2022 was estimated as the proportion of eligible children with documented or recalled receipt of each antigen, using the survey-adjusted methods accounting for sampling weights, clusters and stratification.^2^ KHIS coverage was computed as the total number of doses administered in the aligned window divided by the target population of surviving infants for each antigen. Vaccines administered within health facilities and during outreach sessions were aggregated. Numerators and denominators are provided in (see online supplemental table 1).

Differences in proportions were assessed using a two-proportion z test for descriptive comparison. Absolute percentage-point differences were classified as minimal (<5%), moderate (5–10%) or high (10+%).^27^ These thresholds are consistent with ranges previously applied in immunisation monitoring and data-quality assessments by WHO and United Nations International Children’s Emergency Fund (WUENIC).^28^ Anomalous patterns in KHIS dose counts, we applied Benford’s Law using the mean absolute deviation (MAD); deviations exceeding 0.012 were considered non-conforming.^29^

### Ethical considerations

KDHS data are publicly available and fully anonymised, and their secondary use does not require formal ethical approval or individual participant consent. For the KHIS^21^ dataset, only routinely collected, aggregated, facility-level service statistics (dose administered) were used. No individual patient identifiers or personal data were accessed. Data were obtained under an existing collaboration agreement between the Ministry of Health and the Center for Epidemiological Modelling and Analysis, University of Nairobi. Because the analysis used fully aggregated, non-identifiable administrative data, the requirement for individual informed consent was not applicable and was waived under the terms of the institutional collaboration agreement and applicable national guidelines for secondary use of routine health information system data.

## Results

### National-level comparison

At the national level, vaccine coverage estimates from KDHS 2022 were higher than corresponding KHIS estimates for three of the four antigens assessed, as shown in (table 1). The absolute differences for BCG, pentavalent 1 and MR 1 ranged from approximately 2.4 to 4.5 percentage points and were statistically significant (p<0.001). Pentavalent 3 showed no meaningful difference between the two data sources (0.38 percentage points; p=0.393), indicating close alignment for this antigen. All national-level discrepancies were <5%.

**Table 1:** Overall comparison of vaccine coverage at national level for children 12–23 months old

Vaccine antigen	KDHS 2022 coverage (%)	KHIS coverage (%)	Difference % (95% CI)	P value
BCG	96.92	92.91	4.01 (3.99, 5.13)	<0.001
Pentavalent 1	97.00	92.52	4.48 (3.91, 5.05)	<0.001
Pentavalent 3	90.01	89.63	0.38 (–0.54, 1.42)	0.393
Measles-rubella 1	87.97	85.56	2.41 (1.33, 3.47)	<0.001

The p values were computed and compared with a 5% significance level.

KDHS, Kenya Demographic Health Survey; KHIS, Kenya Health Information System.

### Sub-national comparison

Considerably greater heterogeneity was observed at the county level, as shown in figure 1. Across the 47 counties, table 2 below shows that only 16–21 counties (depending on the antigen) had minimal discrepancies (<5%). Pentavalent 3 demonstrated the highest consistency (44.7% of the counties in the minimal-discrepancy category). BCG and MR 1 showed the poorest agreement, with nearly one-third of counties exhibiting discrepancies exceeding 10%. Pentavalent 1 showed the most pronounced mid-range variability (48.9% of counties with 5%–10% differences).

**Figure 1:**
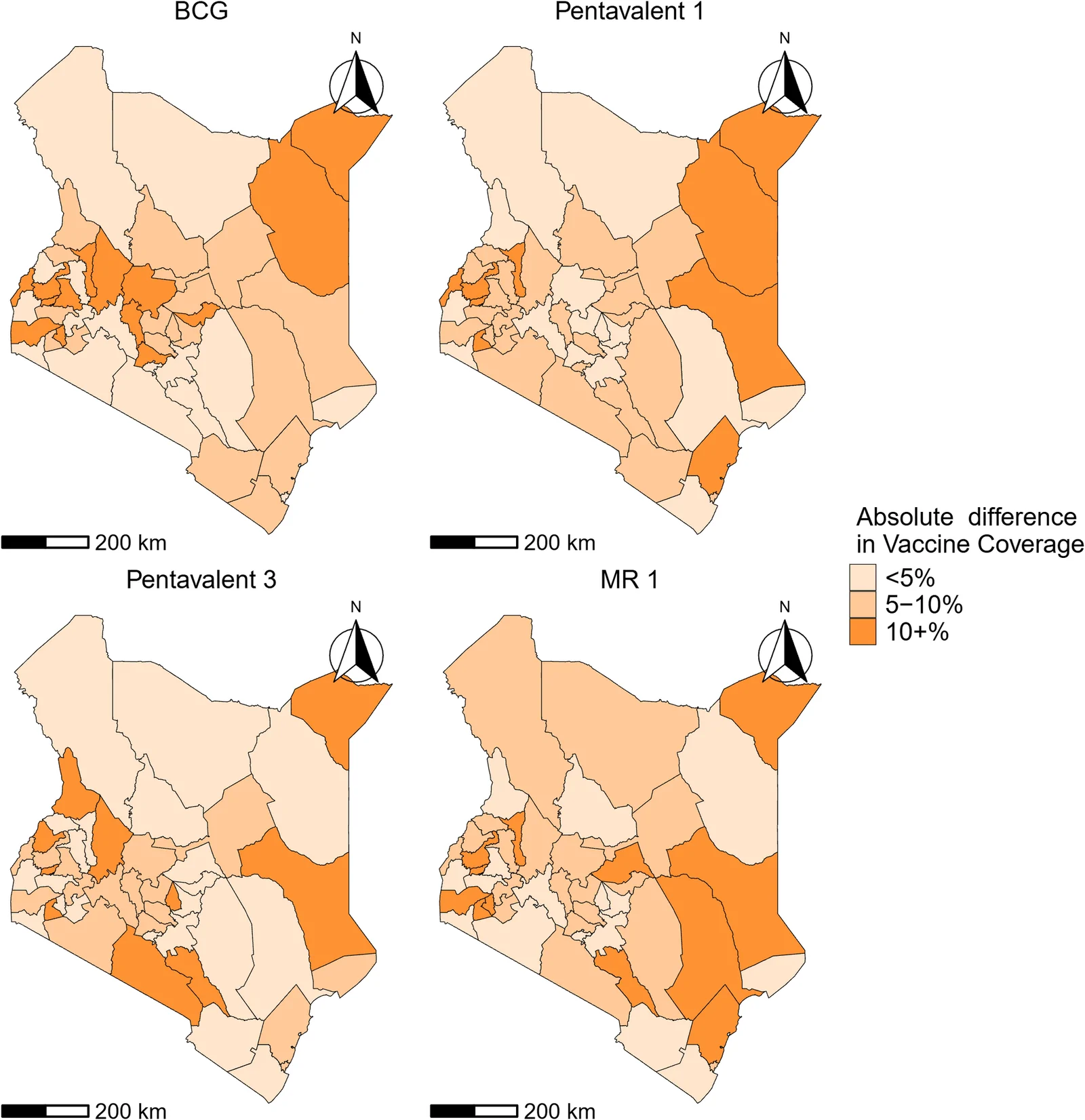
Comparison of discrepancies between the KDHS 2022 and KHIS data sources across counties of Kenya categorised into three levels <5%, 5%–10% and 10+% absolute difference in vaccine coverage. KDHS, Kenya Demographic Health Survey; KHIS, Kenya Health Information System; MR 1, measles-rubella 1.

**Table 2:** Distribution of counties by category of absolute difference in vaccine coverage, in Kenya (n=47)

Absolute difference in vaccine coverage	Number of counties in each category of discrepancy in vaccine coverage (%)			
	BCG	Pentavalent 1	Pentavalent 3	Measles-rubella 1
<5 %	16 (34.0%)	15 (31.9%)	21 (44.7%)	16 (34.0%)
5–10%	17 (36.2%)	23 (48.9%)	16 (34.0%)	19 (40.4%)
10+%	14 (29.8%)	9 (19.1%)	10 (21.3%)	12 (25.5%)

The absolute difference in vaccine coverage was categorised into minimal (<5%), moderate (5–10%) and high (10+%) across the four vaccines (BCG, Pentavalent 1,3 and Measles-rubella 1) with number of counties falling within the three groups of absolute estimate coverage.

Only three of 47 counties (Migori, Siaya and Machakos) had discrepancies of <5% across all four vaccine antigens. In contrast, two counties (Vihiga and Mandera) showed discrepancies >10% across all the vaccine antigens, with Mandera exhibiting the largest overall divergence. Only seven counties had the same discrepancy category for every antigen (see online supplemental table 2).

Figure 2 shows absolute differences in coverage estimates by urban vs rural residence. Differences of >10% were more frequent in rural areas for BCG and measles-rubella 1. For pentavalent 1 and 3, high discrepancies were more common in some eastern and northern counties of Kenya, including Isiolo and Marsabit. These patterns are descriptive only; the analysis did not measure health-system capacity, staffing or access barriers, so they should not be interpreted as evidence of specific systemic causes.

**Figure 2:**
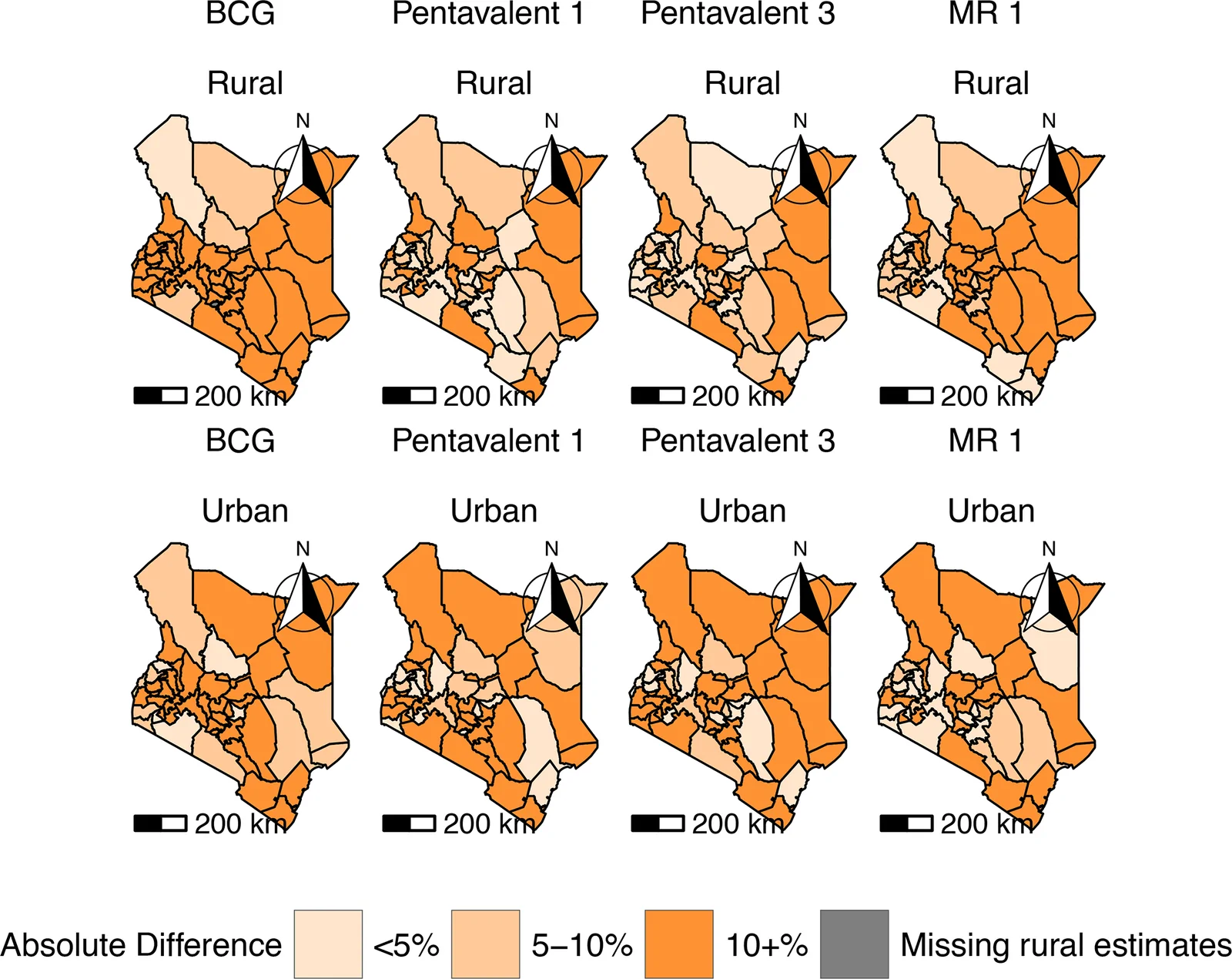
Comparison of discrepancies between the KDHS 2022 and KHIS four vaccine coverage estimates across counties of Kenya categorised into three levels <5%, 5%–10% and 10+% absolute difference by residence which urban and rural with darker shading indicating larger disparities. Mombasa and Nairobi counties were all classified as urban. KDHS, Kenya Demographic Health Survey; KHIS, Kenya Health Information System; MR 1, measles-rubella 1.

## Discussion

This study compared childhood vaccination coverage estimates from KHIS and the 2022 KDHS. National absolute differences were <5% for all four antigens and for pentavalent 3 the difference was not statistically significant. In contrast, fewer than half of counties fell into the <5% discrepancy category for any single antigen and three counties (Migori, Siaya and Machakos) achieved minimal discrepancy across all the four antigens. National concordance therefore does not imply that two data sources are interchangeable for county-level decision-making.

Several factors may contribute to the observed discrepancies, although the study design does not permit direct attribution. Incomplete reporting from private facilities is a plausible contributor: private health facilities account for roughly one-fifth of reporting facilities yet may have weak incentives and motivation^30^ to report free-of-charge immunisation services.^20^ Caregivers recall bias KDHS (24.4% of children lacked vaccination cards) could inflate the survey estimates.^31,32^ Population-denominators misalignment is another persistent challenge; KHIS relies on projected surviving-infant denominators that may not capture local migration or fertility shifts, whereas KDHS uses a weighted sample.^32^ These structural differences increase the likelihood of divergent sub-national estimates even when both systems function as designed.

Antigen-specific patterns are informative. Pentavalent 3 showed the closest national agreement and the highest share of counties with minimal discrepancy. Possible explanations include more consistent reporting for later-dose antigens once children remain in contact with services, compensating biases (under-reporting of doses offset by under-estimated denominators) or differential dropout and missed-opportunity patterns between early- and late-schedule antigens. BCG (often given at birth or first contact) and MR 1 displayed larger and more variable discrepancies, consistent with known challenges in capturing facility births and completing the measles–rubella series. These differences caution against treating any single coverage indicator as representative of overall data quality.

The substantial county level heterogeneity observed in this study underscores variability in both data quality and immunisation service delivery. In Mandera, the omission of one survey cluster during the KDHS data collection may have contributed to distortions in the survey estimate. The consistently wide discrepancies across antigens indicate that routine data quality challenges are also likely. There were no incidents reported in Vihiga that could explain the wide discrepancy in estimates. Similar subnational variation in data has been reported from other low and middle-income countries^33,34^ further highlighting the need for more localised estimates of health indicators where national estimates mask significant sub-national variations.^25,35^

Leveraging routine health information systems as the primary source of immunisation data offers substantial programmatic advantages, including timely performance tracking, rapid identification of coverage gaps and reduced reliance on infrequent and costly large-scale household surveys. At the national level, our findings suggest that KHIS provides sufficiently concordant estimates to support routine monitoring of overall immunisation programme performance. This indicates that, for national reporting and broad trend analysis, KHIS can serve as a practical and efficient monitoring tool.

County-level heterogeneity was observed. In Mandera, omission of one survey cluster during KDHS fieldwork may have contributed to distortion of the survey estimate; no analogous incident was reported for Vihiga county. Similar sub-national variation between routine and survey estimates has been documented in other LMICs, reinforcing the need for localised assessment rather than reliance on national averages.

Temporal alignment was addressed by restricting the KHIS window (March 2020–March 2022) to the birth-cohort range of children aged 12–23 months at the time of the 2022 KDHS. Residual period effects cannot be excluded: the window includes COVID-19-related service disruptions that may have differentially affected facility reporting and actual coverage, as documented in mixed-methods assessments of indirect pandemic effects and KHIS-based monitoring of essential services in Kenya. Any such effects would tend to widen rather than narrow observed differences and do not overturn the central finding of greater sub-national than national discordance, consistent with evidence of large county-level heterogeneity in coverage and poor concordance between survey-modelled and routine estimates at sub-national scales.^36^

Routine systems offer clear advantages—timeliness, lower cost and continuous availability—relative to infrequent household surveys. At national level, KHIS can support broad trend monitoring. However, coverage estimation and programme management are most important at the local (county and sub-county) level. If managers cannot reliably determine coverage in their own jurisdictions, the practical value of aggregated facility-based reporting is substantially diminished.

Two commonly proposed solutions face material constraints. Moving from aggregate tallies to client-linked individual records (as envisaged in KHIS Policy 2020–2030)^37^ would enable tracking of individual trajectories, dropout and timeliness, but requires sustained financing, infrastructure, workforce capacity and data-protection safeguards that remain limited in many counties. More frequent population-based surveys would improve validation capacity, yet their human and financial costs are high, and external support has become less certain. A pragmatic near-term strategy is therefore differentiated: rely on KHIS for national monitoring and in counties that demonstrate stable concordance; prioritise data-quality improvement (completeness audits, private-sector reporting incentives, denominator refinement) in high-discrepancy counties; and retain periodic surveys as an independent validation benchmark rather than as a routine substitute.

The current aggregated KHIS structure also limits the indicators that can be monitored. Because the system records doses rather than unique children, it cannot directly measure full immunisation coverage, defaulter tracing or dose timeliness—core performance metrics for immunisation programmes.

### Limitations of the study

This study had some limitations. KHIS denominators are based on projected surviving-infant populations and may over- or under-estimate coverage at county level. KHIS does not record whether doses were given at the age recommended by the national schedule,^23^ whereas KDHS records all doses regardless of timing. KDHS estimates are subject to recall bias when cards are unavailable.^38^ KHIS reporting may be incomplete, particularly among private providers, and is subject to data-entry errors. The temporal window, while aligned to the relevant birth cohorts, spans a period of health-system disruption whose effects cannot be fully isolated. Despite these limitations, triangulation of the two independent systems provides a useful assessment of concordance and highlights priority areas for strengthening health information systems.

Beyond immunisation, the approach can be extended to other routine indicators such as antenatal-care attendance or skilled birth attendance. Comparable exercises in Mali and Uganda have shown that routine data may support planning at national or well-reporting sub-national levels while diverging substantially elsewhere.^6,39^ Practical steps to strengthen KHIS include automated reporting tools, regular data audits, targeted training, feedback loops and, where feasible, anomaly-detection analytics, prioritised according to the magnitude of observed discordance.

## Conclusion

Kenya’s KHIS and KDHS produce reasonably concordant national-level estimates of childhood vaccination coverage, yet substantial and uneven county-level discrepancies limit the reliability of routine data for sub-national decision-making. The two sources should not be treated as interchangeable at county level. A differentiated monitoring strategy—using KHIS for national trends and in counties with demonstrated concordance while retaining survey validation and targeted data-quality investments where divergence persists—offers a pragmatic path forward under current resource constraints. Improved reconciliation of numerators and denominators, stronger private-sector reporting and gradual movement toward individual-level tracking remain important longer-term goals.

## Supplementary material

10.1136/bmjph-2026-005621 online supplemental table 1

10.1136/bmjph-2026-005621 online supplemental table 2

## Data Availability

Data are available upon reasonable request.
