# Supplementary material for: Comparison of routine health-facility (KHIS) versus house-survey (KDHS) estimates of childhood vaccination coverage in Kenya: a cross-sectional study
Source: BMJ Public Health. 2026 Nov 11;4(4):e005621. doi: 10.1136/bmjph-2026-005621 (PMC13756428; doi:10.1136/bmjph-2026-005621)
Supplement: Supplementary file 1 [file bmjph-4-4-s001.docx]

**Supplementary Table 1: Numerators and denominators used to estimate vaccine coverage from the Kenya Demographic Health Survey (KDHS) and Kenya Health Information System (KHIS)**

| **Vaccine** | **Kenya Demographic Health Survey** | | **Kenya Health Information System** | |
| --- | --- | --- | --- | --- |
|  | **Numerator** | **Denominator** | **Numerator** | **Denominator** |
| BCG | Children 12–23 months who received BCG vaccine | Total number of surveyed children aged 12–23 months who were alive at the time of the survey. | BCG vaccine doses administered from 1 March 2020 to 30 June 2021 which coincides with the months of birth of the youngest and oldest child surveyed in the KDHS. | Estimated population of surviving infants under 1 year during this period. |
| Pentavalent 1 | Children 12–23 months who received pentavalent 1 vaccine. | Total number of surveyed children aged 12–23 months who were alive at the time of the survey. | Health facility records of pentavalent 1 vaccine doses administered between the period between 13 April 2020 and 14 August 2021 which represents the period 6 weeks after the dates of birth of the youngest and oldest child surveyed in KDHS. | Estimated population of surviving infants under 1 year during this period. |
| Pentavalent 3 | Children aged 12–23 months who were alive and received pentavalent 3 vaccine. | Total number of surveyed children aged 12–23 months who were alive at the time of the survey. | Health facility records of pentavalent 3 vaccine doses administered between 6 June 2020 and 7 October 2021 which represents the period 14 weeks after the dates of birth of the youngest and oldest child surveyed in KDHS. | Estimated population of surviving infants under 1 year during this period. |
| Measles Rubella–1 | Children aged 12–23 months who received MR–1 vaccine. | Total number of surveyed children aged 12–23 months who were alive at the time of the survey. | Health facility records of MR–1 vaccine dose administered between 1 December 2020 to 31 March 2022 which represents the period 9 months after the dates of birth of the youngest and oldest child surveyed in KDHS. | Estimated population of surviving infants under 1 year during this period. |

*Note: For incomplete months, the number of vaccines administered was prorated based on the number of days within that month.*
