# Supplementary material for: Comparison of routine health-facility (KHIS) versus house-survey (KDHS) estimates of childhood vaccination coverage in Kenya: a cross-sectional study
Source: BMJ Public Health. 2026 Nov 11;4(4):e005621. doi: 10.1136/bmjph-2026-005621 (PMC13756428; doi:10.1136/bmjph-2026-005621)
Supplement: Supplementary file 2 [file bmjph-4-4-s002.docx]

Supplementary Table 2: Absolute difference in vaccine coverage between KDHS and KHIS estimates, with 95% confidence intervals and p-values, by county.

| County | BCG  Point change | P–value | Pentavalent 1  Point change | P–value | Pentavalent 3  Point change | P–value | MR 1  Point change | P–value |
| --- | --- | --- | --- | --- | --- | --- | --- | --- |
| Baringo | 11%  (8%, 14%) | <0.05 | 10%  (3%, 17%) | <0.05 | 14%  (6%, 22%) | <0.05 | 5%  (-3%, 14%) | 0.240 |
| Bomet | 9%  (7%, 12%) | <0.05 | 8%  (1%, 14%) | <0.05 | 1%  (5%, 8%) | 0.673 | 6%  (-2%, 13%) | 0.150 |
| Bungoma | 2%  (2%, 2%) | <0.05 | 7%  (1%, 13%) | <0.05 | 8%  (3%, 17%) | <0.05 | 8%  (0%, 16%) | <0.05 |
| Busia | 11%  (8%, 14%) | <0.05 | 11%  (4%, 19%) | <0.05 | 6%  (0%, 15%) | <0.05 | 7%  (-2%, 15%) | 0.112 |
| Elgeyo Marakwet | 15%  (13%, 18%) | <0.05 | 12%  (5%, 19%) | <0.05 | 3%  (-5%, 10%) | 0.510 | 12%  (3%, 21%) | <0.05 |
| Embu | 7%  (6%, 7%) | <0.05 | 4%  (-2%, 9%) | 0.179 | 3%  (-4%, 9%) | <0.05 | -2%  (-9%, 6%) | 0.663 |
| Garissa | -8%  (-18%, 2%) | <0.05 | -17%  (-26%, -9%) | <0.05 | -38%  (-46%, -28%) | <0.05 | -39%  (-47%, -31%) | <0.05 |
| Homa Bay | 12%  (9%, 15%) | <0.05 | 9%  (2%, 17%) | <0.05 | 4%  (-4%, 14%) | 0.247 | 13%  (3%, 23%) | <0.05 |
| Isiolo | -6%  (-10%, -3%) | <0.05 | -7%  – | – | -8%  (-11%, -2%) | <0.05 | -8%  (-15%, 1%) | <0.05 |
| Kajiado | -4%  (-8%, 0%) | 0.055 | -9%  – | – | -22%  (-23%, -21%) | <0.05 | -7%  (-14%, 0%) | 0.064 |
| Kakamega | 15%  (15%, 15%) | <0.05 | 11%  (5%, 18%) | <0.05 | 8%  (3%, 17%) | <0.05 | 13%  (5%, 20%) | <0.05 |
| Kericho | 0%  (-6%, 5%) | 0.907 | 8%  (1%, 16%) | <0.05 | 7%  (1%, 17%) | <0.05 | 7%  (-2%, 15%) | 0.119 |
| Kiambu | -12%  – | – | -3%  – | – | -6%  – | – | -9%  – | – |
| Kilifi | 8%  (8%, 8%) | <0.05 | 12%  (4%, 20%) | <0.05 | 8%  (-1%, 18%) | <0.05 | 15%  (5%, 24%) | <0.05 |
| Kirinyaga | -6%  (-9%, -2%) | <0.05 | -3%  – | – | -11%  – | – | -4%  – | – |
| Kisii | 10%  (8%, 13%) | <0.05 | 12%  (3%, 20%) | <0.05 | 10%  (1%, 19%) | <0.05 | 14%  (4%, 24%) | <0.05 |
| Kisumu | 6%  (3%, 10%) | <0.05 | 7%  (2%, 13%) | <0.05 | 2%  (-9%, 4%) | 0.483 | 1%  (-5%, 7%) | 0.782 |
| Kitui | 4%  (-4%, 12%) | 0.309 | 6%  (-2%, 14%) | 0.115 | 3%  (-6%, 11%) | 0.522 | 5%  (-4%, 14%) | 0.266 |
| Kwale | 9%  (5%, 14%) | <0.05 | 4%  (-3%, 11%) | 0.223 | -1%  (-8%, 7%) | 0.890 | 1%  (-7%, 10%) | 0.728 |
| Laikipia | -22%  – | – | -3%  – | – | -6%  (-10%, -2%) | <0.05 | -5%  (-12%, 3%) | 0.207 |
| Lamu | 0%  (-3%, 2%) | 0.863 | 0%  (-3%, 0%) | 0.999 | -7%  (-13%, -2%) | <0.05 | 3%  (-5%, 11%) | 0.418 |
| Machakos | 3%  (-3%, 9%) | 0.330 | 4%  (-3%, 11%) | 0.272 | -2% (-8%, 5%) | 0.640 | -2%  (-8%, 4%) | 0.465 |
| Makueni | 1%  (-3%, 4%) | 0.646 | -6%  – | – | -16%  – | – | -15%  – | – |
| Mandera | -36%  (-44%, -29%) | <0.05 | -41%  – | – | -48%  (-52%, -44%) | <0.05 | -41%  (-47%, -36%) | <0.05 |
| Marsabit | -2%  (-6%, 2%) | 0.380 | -3%  (-8%, 1%) | 0.109 | 3%  (-5%, 10%) | 0.451 | 6%  (-3%, 15%) | 0.227 |
| Meru | 6%  (6%, 7%) | <0.05 | 7%  (0%, 15%) | 0.059 | 4%  (-3%, 12%) | 0.274 | 15%  (5%, 25%) | <0.05 |
| Migori | 3%  (1%, 4%) | <0.05 | 3%  (0%, 7%) | 0.085 | -2%  (-7%, 2%) | 0.317 | 1%  (-5%, 8%) | 0.679 |
| Mombasa | 1%  (-2%, 4%) | 0.551 | 7%  (1%, 14%) | <0.05 | 6%  (-2%, 13%) | 0.127 | 9%  (0%, 17%) | 0.055 |
| Murang'a | 9%  (4%, 15%) | <0.05 | 6%  (-3%, 14%) | <0.05 | 6%  (-3%, 15%) | 0.187 | 5%  (-4%, 14%) | 0.280 |
| Nairobi | 1%  (-2%, 5%) | 0.452 | 6%  (0%, 12%) | <0.05 | 0%  (-6%, 7%) | 0.989 | -1%  (-7%, 5%) | 0.793 |
| Nakuru | 0%  (-2%, 2%) | 0.812 | 3%  (-1%, 7%) | 0.151 | 6%  (1%, 11%) | <0.05 | 1%  (-5%, 6%) | 0.817 |
| Nandi | 16%  (15%, 16%) | <0.05 | 9%  (2%, 17%) | <0.05 | 5%  (1%, 15%) | 0.104 | 2%  (-7%, 12%) | 0.602 |
| Narok | 3%  (1%, 5%) | <0.05 | -5%  – | – | -8%  (-10%, -7%) | <0.05 | 2%  (-6%, 10%) | 0.598 |
| Nyamira | 23%  (22%, 23%) | <0.05 | 9%  (1%, 18%) | <0.05 | -3%  (-7%, 0%) | <0.05 | -11%  – | – |
| Nyandarua | 25%  (24%, 26%) | <0.05 | 9%  (2%, 16%) | <0.05 | 8%  (1%, 15%) | <0.05 | 10%  (2%, 18%) | <0.05 |
| Nyeri | -5%  (-14%, 4%) | 0.244 | 1%  (-7%, 9%) | 0.806 | -6%  (-15%, 2%) | 0.141 | -6%  (-16%, 3%) | 0.205 |
| Samburu | -7%  (-12%, -2%) | <0.05 | -9%  (-10%, -7%) | <0.05 | 1%  (-6%, 7%) | 0.871 | 3%  (-5%, 12%) | 0.438 |
| Siaya | 3%  (1%, 5%) | <0.05 | 4%  (-1%, 9%) | 0.110 | 2%  (-4%, 7%) | 0.536 | 2%  (-4%, 9%) | 0.461 |
| Taita Taveta | 7%  (3%, 11%) | <0.05 | 5%  (-2%, 12%) | 0.145 | -5%  (-12%, 3%) | 0.248 | 3%  (-5%, 12%) | 0.445 |
| Tana River | 7%  (3%, 11%) | <0.05 | -1%  (-5%, 4%) | 0.818 | 1%  (-6%, 9%) | 0.700 | 11%  (-3%, 20%) | <0.05 |
| Tharaka Nithi | 15%  (14%, 15%) | <0.05 | 6%  (0%, 13%) | 0.065 | 5%  (-3%, 13%) | 0.222 | 8%  (-1%, 17%) | 0.085 |
| Trans Nzoia | 5%  (1%, 8%) | <0.05 | 8%  (1%, 14%) | <0.05 | 1%  (-7%, 8%) | 0.841 | 7%  (-1%, 15%) | 0.103 |
| Turkana | 3%  (0%, 6%) | <0.05 | 3%  (-1%, 7%) | 0.176 | 0%  (-6%, 6%) | 0.980 | -5%  (-13%, 3%) | 0.221 |
| Uasin Gishu | 1%  (-1%, 4%) | 0.383 | 8%  (1%, 14%) | <0.05 | 5%  (-2%, 11%) | 0.187 | 6%  (-1%, 13%) | 0.086 |
| Vihiga | 22%  (22%, 23%) | <0.05 | 19%  (8%, 24%) | <0.05 | 17%  (7%, 28%) | <0.05 | 18%  (8%, 28%) | <0.05 |
| Wajir | 22%  (18%, 26%) | <0.05 | 16%  (5%, 19%) | <0.05 | -4%  (-12%, 5%) | 0.389 | 2%  (-6%, 11%) | 0.577 |
| West Pokot | -5%  (-8%, -3%) | <0.05 | 2%  (-2%, 5%) | 0.346 | -13%  (-18%, -8%) | <0.05 | -4%  (-12%, 4%) | 0.337 |

Note: *Values represent the percentage point difference (KDHS*–*KHIS). Positive values indicate higher coverage in KDHS; negative values indicate higher coverage in KHIS. Shading indicates absolute difference: dark shading >10%, medium shading 5–10%, and light shading <5%. The symbol – indicates that the confidence interval or p-value could not be computed because KHIS coverage exceeded 100%. Statistical significance was assessed at α = 0.05. The values in bracket indicate the lower and upper confidence intervals for the difference in estimate coverage.*
